# Home at last III: Transferring *Uechtritzia* and Asian *Gerbera* species into *Oreoseris* (Compositae, Mutisieae)

**DOI:** 10.3897/phytokeys.96.23142

**Published:** 2018-03-08

**Authors:** Xiaodan Xu, Wei Zheng, Vicki A. Funk, Kexin Li, Jie Zhang, Jun Wen

**Affiliations:** 1 Faculty of Art and Communication, Kunming University of Science and Technology, Kunming 650500 China; 2 Faculty of Architecture and City Planning, Kunming University of Science and Technology, Kunming 650500 China; 3 Department of Botany, MRC 166, National Museum of Natural History, Smithsonian Institution, Washington, D.C. 20013-7012 USA

**Keywords:** Compositae, *Gerbera*-complex, *Oreoseris*, *Uechtritzia*, SEM, stomata, pollen, South America, Africa, Asia

## Abstract

Recently the Asian *Gerbera* species were shown to form a clade that was not the sister group of the African *Gerbera*. In this study, the position of the Asian *Gerbera* species was further assessed based on morphology and molecular phylogenetic analyses that included six Asian *Gerbera* and 26 other species from the *Gerbera*-complex. Morphological results showed that the six Asian *Gerbera* species, which were sampled, bear leaves with the adaxial epidermal surface lacking stomates, possess bracteate scapes and lack inner ray florets. These characters suggest that the Asian *Gerbera* species are most closely related to the species of *Uechtritzia*, which also share similar pollen grain size and shape with the Asian *Gerbera*, rather than to the African *Gerbera*. Furthermore, the phylogenetic results based on two nuclear (ITS and ETS) and three chloroplast (*trn*L–*trn*F, *trn*L–*rpl*32 and *trn*C–*pet*N) sequences strongly support the Asian *Gerbera* and *Uechtritzia* forming a clade, with the latter nested within the Asian *Gerbera* species. Both morphological and molecular phylogenetic data thus confirmed the taxonomic identity of the Asian *Gerbera* and *Uechtritzia*. The authors herein formally treat the nine species of the Asian *Gerbera* and the three species of *Uechtritzia* as members of the genus *Oreoseris*, which is the earliest generic name of this lineage and has the nomenclatural priority.

## Introduction

The *Gerbera*-complex (Compositae: Mutisieae) contains eight genera: *Gerbera* L., *Leibnitzia* Cass., *Uechtritzia* Freyn, *Amblysperma* Benth., *Chaptalia* Vent., *Trichocline* Cass., *Perdicium* L. and *Lulia* Zardini. *Gerbera* currently contains about 31 species, which belong to six sections: the five African sections: sect. Gerbera (8 species), sect. Parva H.V.Hansen (1 species), sect. Lasiopus (Cass.) Sch.Bip. (6 species), sect. Pseudoseris (Baill.) C.Jeffrey (8 species, distributed in Madagascar) and sect. Piloselloides Less. (2 species, one of which is widespread in Asia and Africa) and the Asian sect. Isanthus (Less.) Jeffrey (6 species; Hansen 1985a, 1985b, 1988, Johnson et al. 2014, Funk et al. 2016). One South American species *G. hieracioide*s (Kunth) Zardini was not included in any of the above-mentioned sections of *Gerbera* (Zardini 1974) and the authors have recently transferred it to *Chaptalia* based on both morphological and molecular data (Xu et al. 2018).

The Asian Gerbera section Isanthus is characterised mainly by campanulate involucres, naked receptacles and rostrate achenes (Hansen 1988). A recent molecular phylogenetic analysis showed that the Asian *Gerbera* species did not form a clade with the African species (Pasini et al. 2016): the Asian *Gerbera* + *Uechtritzia* formed a clade and the African *Gerbera* and *Amblysperma* constituted another clade. Some earlier workers also suggested treating the Asian section as an entity separate from the African *Gerbera* (Candolle 1838, Jeffrey 1967). Hansen (1990), however, argued that, while the Asian Gerbera sect. Isanthus differed somewhat from the African *Gerbera*, it shared four apomorphies as well as 11 plesiomorphies with *Uechtritzia* and the three entities could not be discerned from one another.

Species of *Uechtritzia* have hemispherical involucres, fimbriate receptacles and slightly rostrate achenes (Hansen 1988). This genus contains three species, namely *U. armena* Freyn endemic to Turkey (Doganet al. 2016) and Armenia, *U. kokanica* (Regel et Schmalh.) Pobed. from Central Asia (Kazakhstan, Uzbekistan, Tajikistan, Kyrgyzstan, Turkmenistan to Afghanistan) and *U. lacei* (G.Watt) C.Jeffrey of the Himalayan region (Hansen 1988).

Pasini et al. (2016) included one *Uechtritzia* species, *U. kokanica* and showed that the species was nested within the two sampled Asian *Gerbera* species based on nuclear (ITS) and chloroplast (*trn*L–*trn*F) sequence data. This result indicated the possibility that the Asian *Gerbera* may belong to the genus *Uechtritzia*. However, the phylogenetic position and the taxonomic identity of the Asian *Gerbera* need to be tested with an expanded taxon sampling by adding more Asian and African species of *Gerbera* and *Uechtritzia* before any taxonomic decisions can be made.

In this study, the phylogenetic position of the Asian *Gerbera* was tested by expanding the taxon sampling of the Asian and African *Gerbera* and the *Uechtritzia* species and using both molecular (two nuclear markers: ITS and ETS and three chloroplast markers: *trn*L–*trn*F, *trn*L–*rpl*32 and *trn*C–*pet*N) and morphological data (leaf adaxial surface, pollen, scape and floral morphology).

## Materials and methods

A total of 32 species from eight genera of the *Gerbera*-complex and *Adenocaulon chilense* (outgroup) were sampled for this study (Tables 1, 2). The morphological data were taken from specimens at the United States National Herbarium (US) and included characters of the leaf epidermis, pollen, flowers and scapes.

**Adaxial leaf epidermal and pollen morphology.** A small area of the leaf lamina (about 0.5–1.0 cm^2^) was placed with the adaxial side exposed, on carbon tape over stubs for the scanning electron microscopy (SEM). For the pollen analysis, samples were dehydrated and were then placed on aluminium stubs using double-sided adhesive tape following Wen and Nowicke (1999). The stubs bearing the leaf sample and pollen were treated with gold-palladium to 16.6 μm thickness and were examined under a Philips XL-30 scanning electron microscope at the SEM Lab of the National Museum of Natural History (NMNH), Smithsonian Institution. The 22 samples were subsequently observed and photographed under the SEM using the proprietary software associated with the Philips SEM. Images of at least 15 different areas of the adaxial leaf surface were captured for each sample, as well as 20 pollen grains. The polar and equatorial axes of pollens were measured by ImageJ 1.8.0.

**DNA extraction, amplification and sequencing.** The DNA molecular work was undertaken in the Laboratory of Analytical Biology (LAB) of NMNH. DNA from 16 samples (15 species) was extracted through AutoGen (AutoGen Inc., Holliston, Massachusetts, USA) or the DNeasy Plant Mini Kit (Qiagen, Valencia, California, USA). Leaf tissue samples, along with 1.0 and 2.3 mm diameter beads, were dipped in liquid nitrogen then immediately shaken for 60 seconds at 1800 rpm by Tissuelyser. About 500 µl of the CTAB extraction buffer was added to the tubes, vortexed and incubated overnight (500 rpm at 55 °C). Then 300 µl of the supernatant was transferred to an AutoGen plate. The AutoGen was run according to the manufacturer’s default settings.

Five markers including two nuclear ribosomal ITS and ETS and three chloroplast *trn*L–*trn*F, *trn*L–*rpl*32 and *trn*C–*pet*N intergenic spacers were amplified. The ITS primers were designed by Downie and Katz-Downie (1996) and White et al. (1990), ETS primers by Baldwin and Markos (1998); *trn*L–*trn*F primers by Taberlet et al. (1991), *trn*L–*rpl*32 spacer primers by Timme et al. (2007) and *trn*C–*pet*N spacer primers by Lee and Wen (2004) (Table 3). The PCR reaction mixture had a total of 25 µl volume, comprising 14.05 µl nuclease free water, 2.5 µl 10x buffer, 2 µl dNTPs, 1.25 µl MgCl_2_, 1 µl of both forward and reverse primers, 0.5 µl BSA, 0.2 µl Taq DNA polymerase and 2.5 µl of template DNA. The PCR reactions were performed in a Veriti PCR Thermal Cycler. The amplification protocols for all markers are summarised in Table 3. The amplified products were purified with ExoSapIT enzyme with activation at 37 °C and deactivation at 95 °C. 4 µl of the purified product and same primers (1 µl, 1 µM) were cycle-sequenced in a mixture containing 0.8 µl Big Dye (Applied Biosystems, Foster City, California, USA) and 2.0 µl 5x Big Dye buffer and 4.2 µl water.

The cycle sequencing programme was 30 cycles of 95 °C for 30 s, 50 °C for 30 s and 60 °C for 4 min. The resultant product was sephadex-filtered and sequenced through an ABI 3730 automated sequencer (Applied Biosystems, Foster City, USA). Sequences were aligned by using MAFFT (Katoh and Standley 2013) in Geneious 10.0.9. (Biomatters Ltd., Auckland, New Zealand) and checked manually. A total of 90 newly generated sequences from the 23 samples were deposited in GenBank (Table 2).

A total of 16 sequences of eight species were retrieved from NCBI for the related taxa within the tribe Mutisieae (Table 2). Phylogenetic relationships were inferred based on the concatenated ITS+ETS+*trn*L–*rpl*32+*trn*L–*trn*F+*trn*C–*pet*N data with MrBayes v. 3.2.2 (Ronquist et al. 2012) by using the substitution model of GTR based on the best-fitting model determined by jModelTest 2.1.6 (Posada 2008), the chain length of 10,000,000, rate variation of gamma, gamma categories of 4, heated chains of 4, heated chain temp of 0.2, subsampling freq. of 200 and burn-in length of 100,000. Tracer v. 1.5 (Rambaut and Drummond 2009) was used to confirm that the effective sample size (ESS) for all relevant parameters was > 200. After discarding the trees as burn-in, a 50 % majority-rule consensus tree and posterior probabilities (PP) for node support were calculated using the remaining trees.

## Results

**Figure 1.**
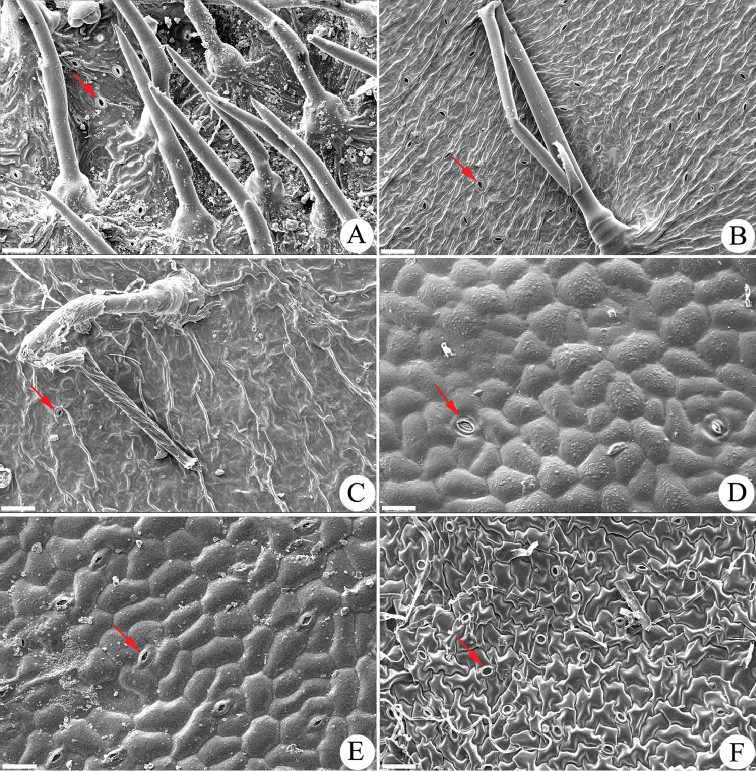
Adaxial leaf epidermal surface morphology of African *Gerbera* and Asian *Leibnitzia*. **A** *G. ambigua* (sect. Lasiopus) **B** *G. piloselloides* (sect. Piloselloides) **C** *G. perrieri* (sect. Pseudoseris) **D** *G. serrata* (sect. Gerbera) **E** *G. crocea* (sect. Gerbera) **F** *L. nepalensis.* Bar=50 μm.

**Figure 2.**
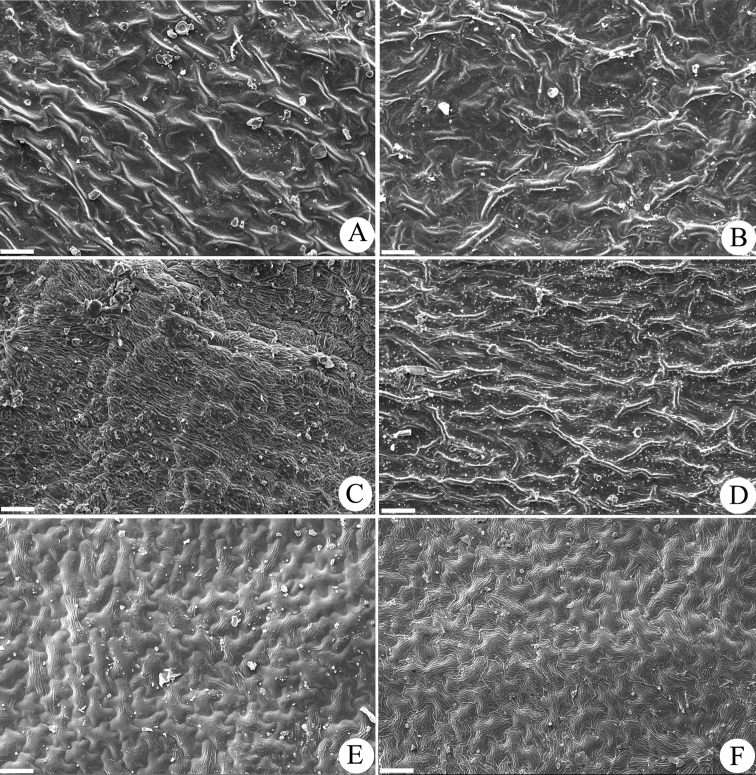
Adaxial leaf epidermal surface morphology of Asian *Gerbera* and *Uechtritzia*. **A** *G. maxima* **B** *G. delavayi* **C** *G. gossypina* **D** *G. nivea* **E** *U. kokanica* **F** *U. lacei.* Bar=50 μm.

**Adaxial leaf epidermal morphology.** The results of the SEM work (Table 1) showed that the six tested Asian *Gerbera* species have no stomates on the adaxial leaf surface (Figure 2A, B, C, D). This adaxial leaf morphological trait differs from that of the African *Gerbera* species: (1) Three East African *Gerbera* sections sampled [sect. Lasiopus (4 species), sect. Piloselloides (2 species) and sect. Pseudoseris (2 species)] have stomates and stiff, straight, upright trichomes on the adaxial surface. Figure 1 has representative images for each of the above sections: *G. ambigua* (Fig. 1A), *G. piloselloides* (Fig. 1B) and *G. perrieri* (Fig. 1C), respectively. (2) Members of the South African sect. Gerbera have stomates. Five species were examined and the epidermal characters are represented by *G. serrata* (Fig. 1D) and *G. crocea* (Fig. 1E). Furthermore, the adaxial leaf morphological traits of the Asian *Gerbera* species also deviate from two Asian-American disjunct *Leibnitzia* species, which have stomates on the adaxial leaf epidermal, as represented by *L. nepalensis* (Fig. 1F). Nevertheless, the Asian *Gerbera* samples share similar adaxial leaf epidermal characters of lacking stomates with the two examined *Uechtritzia* species, *U. kokanica* (Fig. 2E) and *U. lacei* (Fig. 2F). Based on the adaxial leaf epidermal morphology, the Asian *Gerbera* is most closely related to *Uechtritzia* rather than to the African *Gerbera*.

**Table 1.** Voucher information and morphological characters of *Gerbera* and related species.

Species	Section	Locality	Voucher information	Adaxial leaf stomata	Bracts on scape	Inner rays	Pollens	
							Polar axis (µm)	P/E ratio
*Gerbera viridifolia* (DC.) Sch.Bip.	*Lasiopus*	Kenya	*T.H. Trinder-Smith s.n.* (US)	+	−	+	44.12	1.21
*G. jamesonii* Adlam	*Lasiopus*	Cultivar	*T. Derby s.n.* (US)	+	−	+	45.77	1.29
*G. aurantiaca* Sch.Bip.	*Lasiopus*	South Africa	*Bayliss 2505* (US)	+	−	+	43.48	1.20
*G. ambigua* Sch.Bip.	*Lasiopus*	South Africa	*M. Koekemoer 2097* (US)	+	−	+	44.98	1.38
*G. piloselloides* Cass.	*Piloselloides*	Swaziland	*M. Koekemoer 2590* (US)	+	−	+	42.09	1.28
*G. cordata* Less.	*Piloselloides*	Madagascar	*T.B. Croat 29083* (MO)	+	−	+	43.19	1.27
*G. perrieri* Humbert	*Pseudoseris*	Madagascar	*L. Gautier 3110* (MO)	+	−	+	44.04	1.29
*G. diversifolia* Humbert	*Pseudoseris*	Madagascar	*B. Lewis 1201* (MO)	+	−	+	45.31	1.20
*G. crocea* Kuntze	*Gerbera*	South Africa	*M. Koekemoer 2029* (US)	+	+	−	53.83	1.39
*G. linnaei* Cass.	*Gerbera*	South Africa	*E. Werdermann 749* (US)	+	+	−	47.01	1.25
*G. tomentosa* DC.	*Gerbera*	South Africa	*P. Bond 745* (US)	+	+	−	50.43	1.26
*G. wrightii* Harv.	*Gerbera*	South Africa	*P. Goldblatt 5287* (US)	+	+	−	N	N
*G. serrata* Druce	*Gerbera*	South Africa	*M. Koekemoer 2001* (PRE)	+	+	−	N	N
*G. gossypina* Beauverd	*Isanthus*	India	*W.N. Koelz 4828* (US)	−	+	−	50.05	1.40
*G. maxima* Beauverd	*Isanthus*	India	*D.H. Nicolson* 2755 (US)	−	+	−	50.41	1.26
*G. delavayi* Franch.	*Isanthus*	China	*X. Xu 1102* (KMUST)	−	+	−	51.90	1.27
*G. nivea* Sch.Bip.	*Isanthus*	China	*J.F. Rock 6430* (US)	−	+	−	50.30	1.39
*G. raphanifolia* Franch.	*Isanthus*	China	*J.F. Rock 10504* (US)	−	+	−	51.74	1.28
*G. henryi* Dunn	*Isanthus*	China	*W.B. Hemsley 1903* (US)	−	+	−	51.91	1.33
*Uechtritzia armena* Freyn	N	Turkey	*A. Kaya 1835* (EU)	N	+	−	N	N
*U. lacei* (G.Watt) C.Jeffrey	N	India	*W. Koelz 8710* (NA)	−	+	−	50.86	1.36
*U. kokanica* (Regel et Schmalh.) Pobed.	N	Tajikistan	*F.L. Zaprjagaev 4682* (US)	−	+	−	55.80	1.31
*Leibnitzia anandria* (L.) Nakai	N	China	*I. Thomas 8183* (US)	+	+	−	34.45	1.10
*L. nepalensis* (Kunze) Kitam.	N	China	*J. Wen 542* (US)	+	+	−	32.16	1.20
*L. occimadrensis* G.L.Nesom	N	Mexico	*H.S. Gentry 7189* (US)	+	+	−	37.33	1.16
*Amblysperma scapigera* Benth.	N	Australia	*A. Morrison s.n.* (US)	+	+	−	51.60	1.17
*A. spathulata* (A.Cunn. ex DC.) D.J.N.Hind	N	Australia	*R.A. Davis 8267* (US)	+	+	−	55.10	1.23

Notes: + designates those mentioned present; − designates those mentioned absent; N represents data not available.

**Table 2.** Voucher information and GenBank accessions of *Gerbera* and the related species.

Species	Locality	Voucher information	ITS	ETS	trnL–trnF	trnL–rpl32	trnC–petN
*Gerbera viridifolia* (DC.) Sch.Bip.	South Africa	*T.H. Trinder-Smith s.n.* (US)	MG661696*	MG661588*	MG661639*	MG661670*	MG661628*
*G. crocea* Kuntze	South Africa	*M. Koekemoer 2029* (US)	MG661709*	MG661606*	MG661645*	MG661683*	MG661618*
*G. delavayi* Franch.	China	*X. Xu 1102* (KMUST)	MG661708*	MG661605*	MG661659*	MG661682*	MG661619*
*G. henryi* Dunn	China	*X. Xu 1103* (KMUST)	MG661706*	MG661602*	MG661655*	MG661681*	MG661621*
*G. nivea* Sch.Bip.	China	*Y.S. Chen 2674* (PE)	MG661703*	MG661598*	MG661648*	MG661678*	N
*G. aurantiaca* Sch.Bip.	South Africa	*Bayliss 2505* (US)	MG661711*	MG661610*	MG661637*	MG661687*	MG661615*
*G. ambigua* Sch.Bip.	South Africa	*M. Koekemoer 2097* (US)	MG661712*	MG661611*	MG661636*	MG661688*	N
*G. jamesonii* Adlam	Cultivar	*T. Derby s.n.* (US)	MG661704*	MG661599*	MG661638*	MG661679*	MG661624*
*G. cordata* Less.	South Africa	*J. Wen 10067* (US)	N	MG661608*	MG661661*	MG661685*	MG661617*
*G. piloselloides* Cass.	Swaziland	*M. Koekemoer 1972* (US)	MG661701*	MG661592*	MG661650*	MG661675*	MG661625*
*G. wrightii* Harv.	South Africa	*P. Goldblatt 5287* (US)	MG661695*	MG661587*	MG661642*	N	N
*G. serrata* Druce	South Africa	*M. Koekemoer 2001* (PRE)	MG661697*	MG661590*	MG661656*	MG661671*	N
*G. diversifolia* Humbert	Madagascar	*B. Lewis 1201* (MO)	N	MG661604*	MG661640*	N	N
*G. raphanifolia* Franch.	China	*Rock JF 10504* (US)	N	N	MG661658*	N	MG661626*
*G. gossypina* Beauverd	India	*W.N. Koelz 4824* (US)	MG661707*	MG661603*	MG661646*	N	MG661620*
*G. maxima* Beauverd	India	*F. Kingdom-Ward 18199* (NY)	KX349402	N	KX349371	N	N
*Uechtritzia lacei* (G.Watt) C.Jeffrey	India	*W. Koelz 8710* (NA)	N	N	MG661644*	N	N
*U. kokanica* (Regel & Schmalh.) Pobed.	Tajikistan	*F.L. Zaprjagaev 4682* (US)	N	MG661580*	MG661643*	N	MG661635*
*U. kokanica* (Regel & Schmalh.) Pobed.	Tajikistan	*Zaprjagaev s.n.* (NY)	KX349400	N	KX349401	N	N
*Amblysperma scapigera* Benth.	Australia	*A. Morrison s.n.* (US)	MG661713*	MG661612*	N	MG661689*	N
*A. spathulata* (A.Cunn. ex DC.) D.J.N.Hind	Australia	*Cranfield 16197* (CANB)	JX564767	N	KF989620	N	N
*Adenocaulon chilense* Less.	Chile	*G.L. Sobel 2558* (US)	MG661714*	N	N	MG661690*	N
*Chaptalia pringlei* Greene	Mexico	*G. Nesom 4405* (US)	GU126773	N	N	N	N
*C. hieracioides* (Kunth) X.-D.Xu & W.Zheng	Ecuador	*P.M. Peterson 9287* (US)	MG661705*	MG661601*	MG661657*	MG661680*	N
*Trichocline reptans* (Wedd.) Hieron	Argentina	*E. Pasini & F. Torchelsen 1025* (ICN)	KX349398	N	KX349399	KX349410	N
*Leibnitzia anandria* (L.) Nakai	China	*I. Thomas 8183* (US)	MG661694*	MG661585*	MG661662*	MG661668*	MG661629*
*L. anandria* (L.) Nakai	Japan	*Z.Y. Wu 8985* (KUN)	MG661692*	MG661584*	MG661664*	MG661667*	MG661631*
*L. occimadrensis* G.L.Nesom	Mexico	*H.S. Gentry 7189* (US)	GU126784	MG661583*	N	MG661666*	MG661632*
*L. nepalensis* (Kunze) Kitam.	China	*J. Wen 542* (US)	KX349373	MG661582*	KX349374	GU126759	MG661633*
*L. lyrata* (Sch.Bip.) G.L.Nesom	USA	*G. Nesom 24778* (ARIZ)	GU126779	N	N	GU126757	N

Notes: * designates the new sequences from this study; N represents data not available.

**Table 3.** Amplification protocols for all markers.

Marker	Primers and sequences 5'–3'	PCR protocol: initial pre-heating; DNA denaturation; primer annealing; DNA extension; final extension
ITS	ITS5A: GGAAGGAGAAGTCGTAACAAGGITS4: TCCTCCGCTTATTGATATGC	95 °C 1 min; 54 °C 1 min; 72 °C 1 min; 72 °C 10 min; 35 cycles
ETS	18s-ETS: ACTTACACATGCATGGCTTAATCTETS-Hel-1: GCTCTTTGCTTGCGCAACAACT	94 °C 0:30 min; 60 °C 0:40 min; 72 °C 1:20 min; 72 °C 5 min; 30 cycles
*trn*L–*trn*F	*trn*L-Fc: CGAAATCGGTAGACGCTACG*trn*L-Ff: ATTTGAACTGGTGACACGAG	94 °C 1 min; 53 °C 1 min; 72 °C 2 min; 72 °C 10 min; 35 cycles
*trn*L–*rpl*32	*trn*L: TACCGATTTCACCATAGCGG*rpl*32: AGGAAAGGATATTGGGCGG	95 °C 3 min; 51 °C 40 s; 72 °C 1:20 min; 72 °C 5 min; 35 cycles
*trn*C–*pet*N	*trn*C: CCAGTTCAAATCTGGGTGTC*pet*N: GGATATAGTAAGTCTTGCTTGGG	95 °C 3 min; 54 °C 45 s; 72 °C 1:20 min; 72 °C 8 min; 35 cycles

**Figure 3.**
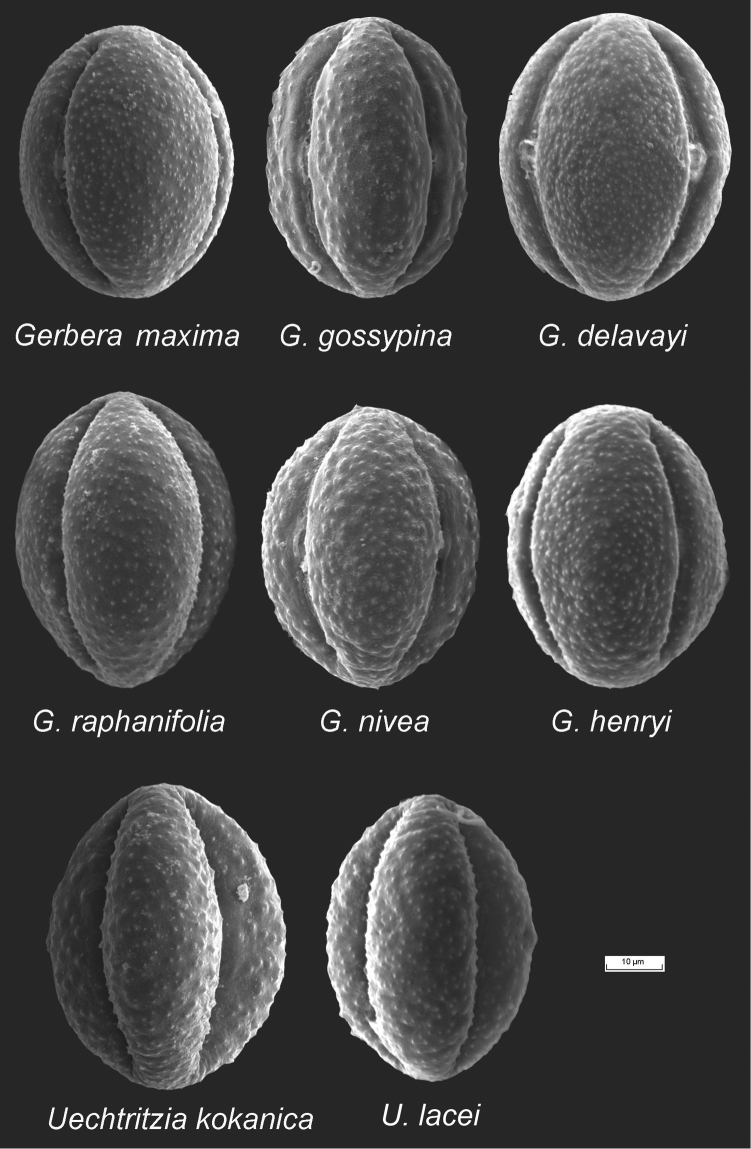
Pollen morphology of Asian *Gerbera* and *Uechtritzia*.

**Figure 4.**
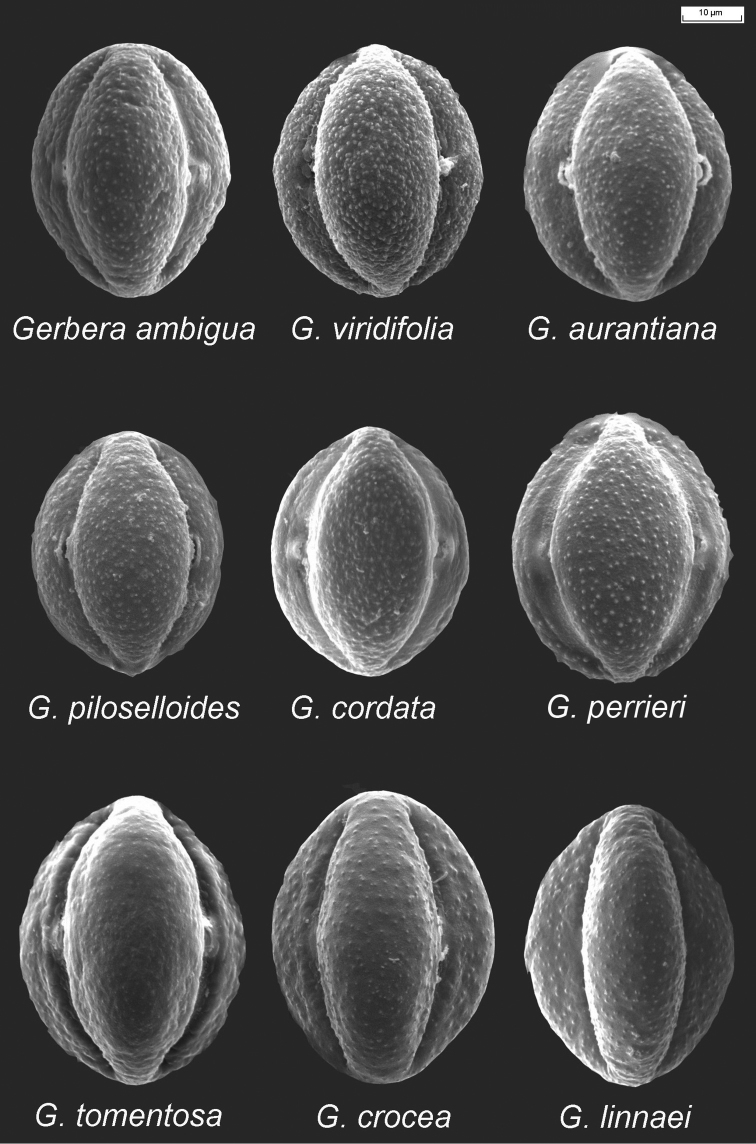
Pollen morphology of African *Gerbera* species.

**Figure 5.**
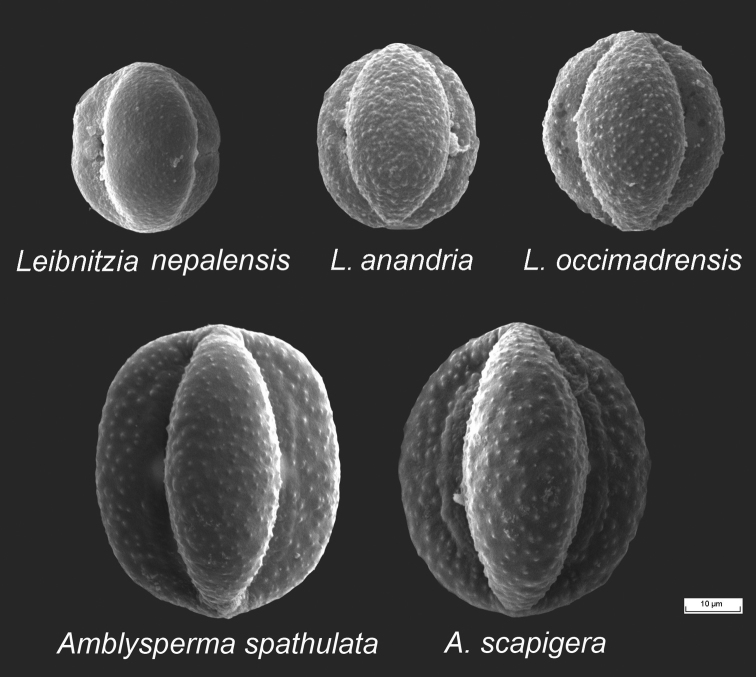
Pollen morphology of *Leibnitzia* and *Amblysperma* species.

**Pollen morphology.** The pollen grains of the examined species of the *Gerbera*-complex are very similar to one another, differing only in the size of the grains as well as the granules on the surfaces (Figs 3 and 4). They are tricolporate, have a granule exine and are prolate and subprolate in shape. The ratios of the polar axis and equatorial axis (P/E) are given in Table 1. For *Gerbera* and *Uechtritzia*, the P/E ratios are between 1.2–1.4. The average polar axis of the Asian *Gerbera* and *Uechtritzia* pollen grains is 50.05–55.80 µm. For the African *Gerbera*, however, the average polar axis of pollen grains is 42.09–45.77 µm in sects. *Lasiopus*, *Piloselloides* and *Pseudoseris* and 47.01–53.83 µm in sect. Gerbera. The P/E ratio of the pollen grains of the Asian *Gerbera* and *Uechtritzia* (Table 1) differs from that of the East Asian-North American *Leibnitzia* and the Australian *Amblysperma*, which fall between 1.10–1.20 (Fig. 5). Furthermore, the average polar axis of the Asian *Gerbera* and *Uechtritzia* pollen grains is higher than that of *Leibnitzia* species, which has the range of 32.16–37.33 µm.

**Figure 6.**
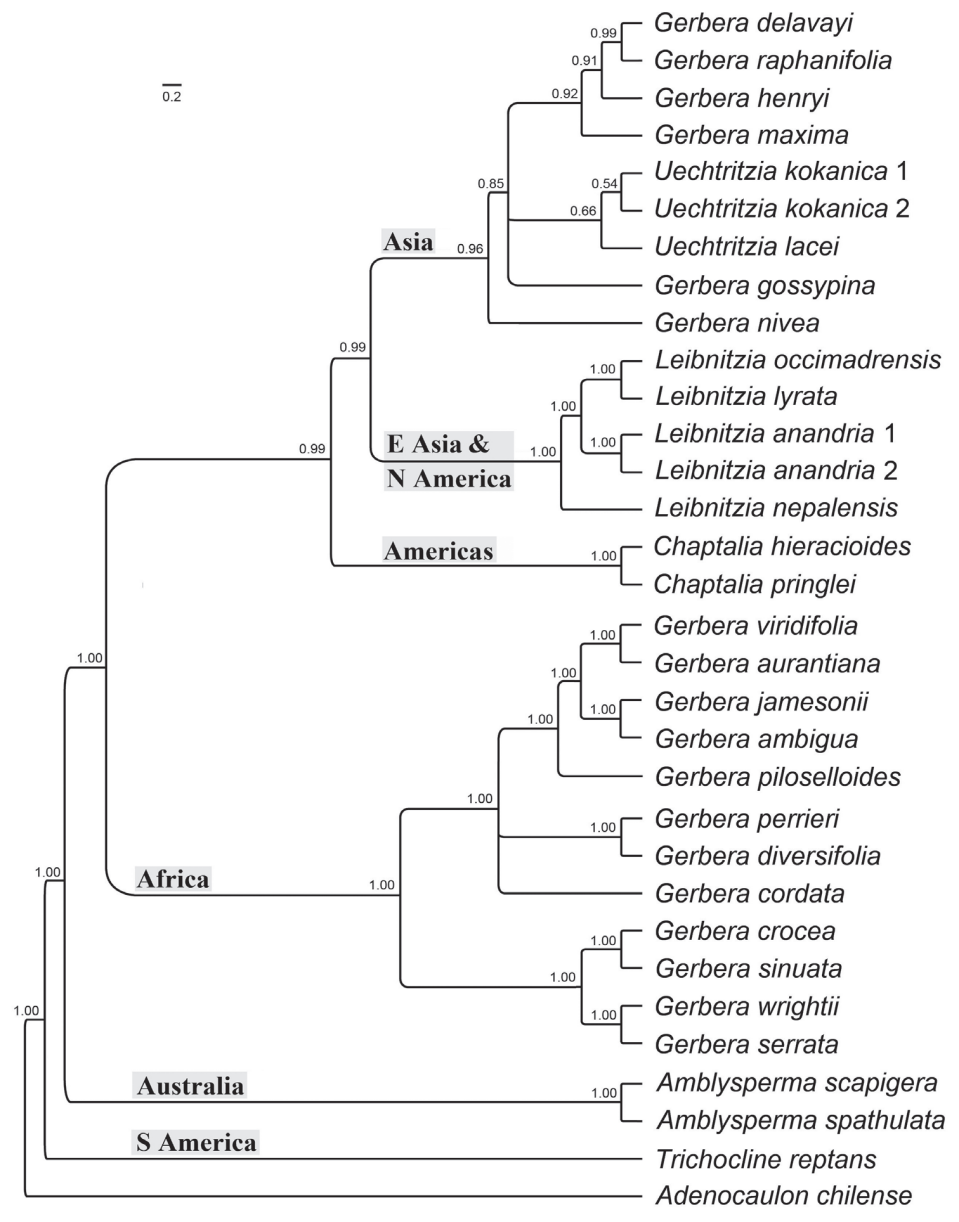
Phylogeny of the *Gerbera*-complex. The phylogeny is based on the Bayesian inference of the combined ITS and ETS, *trn*L–*trn*F, *trn*L–*rpl*32 and *trn*C–*pet*N markers. The posterior probabilities are shown next to branches.

**Phylogenetic analysis.** The Bayesian analysis of the combined nuclear markers and three plastid genes showed six clades of the sampled species of the *Gerbera*-complex, all showing a strong geographic signal (Fig. 6): (1) the Asian *Gerbera* and the *Uechtritzia* species, (2) the East Asian and North American *Leibnitzia* species, (3) the New World genus *Chaptalia*, (4) the African *Gerbera* species, (5) the Australian genus *Amblysperma* and (6) the South American genus *Trichocline*. The three samples of *Uechtritzia* (two species of *U. kokanica* and *U. lacei*) were clearly nested within the Asian *Gerbera* clade (Fig. 6).

## Discussion

Based on this study, the Asian *Gerbera* and the *Uechtritzia* species share several morphological characters, including bracteate scapes, absence of inner ray florets, no stomates on the adaxial leaf surface and similar pollen size and shape (Table 1). Hansen (1990) also commented that the Asian *Gerbera* (i.e. sect. Isanthus) is morphologically similar to *Uechtritzia* and presented a key to distinguish Gerbera sect. Isanthus, *Uechtritzia* and *Leibnitzia*. The differences between the Asian Gerbera sect. Isanthus and *Uechtritzia* were minor. *Uechtritzia* species generally have hemispherical heads, alveolar receptacles that are fimbriate-ciliate; margins of involucral bracts (at least the upper part) often with reddish hairs; achenes that are slightly or indistinctly tapering with hairs that are long-villose, ca. 1 mm long (Katinas 2004) and sericeous (Hansen 1988). Gerbera sect. Isanthus has heads campanulate; a receptacle that is alveolate and naked; the margins of involucral bracts are without reddish hairs; the achenes are tapering and pilose glabrous, with hairs that are shorter, tapered and not sericeous (Hansen 1988). The heads of *Uechtritzia* were reported as hemispherical, in contrast to the heads of the Asian Gerbera sect. Isanthus which are campanulate. However, the species of *U. armena* (the type species of the genus) from Turkey showed the heads as campanulate in the fresh plants (Dogan et al. 2016), which is the same as the Asian Gerbera sect. Isanthus (e.g. *G. delavayi*; Zheng et al. 2017).

Some previous workers argued that the species of Asian Gerbera (sect. Isanthus) should be treated as an entity, separate from African *Gerbera* (Candolle 1838, Jeffrey 1967, Pasini et al. 2016). The results presented here show that the Asian Gerbera sect. Isanthus differs from the African Gerbera sect. Lasiopus, sect. Piloselloides and sect. Pseudoseris in the ebracteate scapes, presence of inner ray florets, stomates on the adaxial leaf surface and smaller pollen size of the African *Gerbera* compared with the Asian *Gerbera*. Although the Asian Gerbera sect. Isanthus shares the traits of bracteates scapes, absence of inner ray florets and similar pollen size with Gerbera sect. Gerbera, the Asian species have no stomates on the adaxial leaf surface. Hansen (1990) stated that the Asian Gerbera sect. Isanthus shows style-arms laterally dilated and truncate achenes; in contrast, the African Gerbera sect. Gerbera has the style-arm slender and achenes tapering or beaked. Additionally, most species of the African Gerbera sect. Gerbera grow in open areas, have leathery leaves and flower only in the spring and summer (Manning et al. 2016), whereas the Asian species of Gerbera sect. Isanthus often grow in forest habitats, have herbaceous leaves and flower in the winter (Gao et al. 2011).

The two *Uechtritzia* species sampled in the molecular phylogeny (Fig. 6) were nested within the Asian *Gerbera* species based on two nuclear markers (ITS and ETS) and three chloroplast markers (*trn*L–*trn*F, *trn*L–*rpl*32 and *trn*C–*pet*N). This result, based on the authors’ expanded taxon and character sampling, is consistent with the findings of Pasini et al. (2016). This study included two of the three species of *Uechtritzia* (Hansen, 1988) and six of the nine Asian Gerbera sect. Isanthus taxa (Gao et al. 2011). The phylogenetic analysis clearly supports the species of *Uechtritzia* as nested within the Asian *Gerbera* and this clade is the sister group of *Leibnitzia* with strong support (PP=0.99) (Fig. 6).

*Leibnitzia* is a genus containing about six species with a disjunct distribution: four species in Asia (Gao et al. 2011) and two species in Mexico (Baird et al. 2010). It shows the same characters of bracteate scapes and no inner ray florets as the Asian *Gerbera* + *Uechtritzia*. It differs from the latter by the presence of stomates on the adaxial leaf surface and smaller pollen size (polar axis of 32.16–37.33 µm) compared with Asian *Gerbera* + *Uechtritzia* (polar axis of 50.05–55.80 µm). Furthermore, *Leibnitzia* has two generations of heads (a vernal generation with chasmogamous capitula and an aestival generation with cleistogamous capitula), subseriate involucral bracts, slender style-arm, anthers of the ray flowers reduced to threads or wanting and achenes that are tapering or beaked. The Asian *Gerbera* + *Uechtritzia*, on the other hand, have one generation of heads, imbricate involucral bracts, laterally dilated style-arms, a fully developed apex (and base) on the anthers in the ray flowers and truncate achenes (Hansen, 1990).

Based on the molecular phylogenetic results, the Asian *Gerbera* species are closest to *Uechtritzia*, with the latter nested within the Asian *Gerbera* species. *Leibnitzia* shows significant morphological differences to the *Asian Gerbera + Uechtritzia*. The taxonomic identity of *Uechtritzia* and the Asian *Gerbera* is strongly supported by the morphology of inflorescences, scapes, capitula, pollen and the lack of stomates on the adaxial leaf surface. Therefore, the authors herein include the nine Asian *Gerbera* species and the three *Uechtritzia* species in *Oreoseris* DC. which is the earliest available name for the expanded Eurasian genus.

## Taxonomic synopsis with nomenclatural changes

In trying to determine the correct genus name for the Eurasian clade, it is necessary to investigate three relevant generic names. *Gerbera* L. was described in 1758; *Arnica gerbera* L. is the basionym of the African species *G. linnaei* Cass., the conserved type of *Gerbera* L. (lectotype designated by Hansen 1985a). *Gerbera* was named after Traugott Gerber, a German naturalist who died in 1743. *Oreoseris* DC. was described in 1838 and its type species is *O. nivea* DC. which was designated by Hansen in 1988. While de Candolle (1838) did not say why he named the genus, *Oreo* is from the Greek *oreos* for mountain and, in his description, de Candolle says that the genus is a “…perennial herb from the mountains of eastern India (translated).” *Uechtritzia* Freyn was described in 1892; the type species is *U. armena* Freyn (lectotype designated by Pobedimova, 1963). The genus was named in honour of Rudolf Karl Friedrich von Uechtritz (1838–1886), a botanist from Wroclaw, Poland (ex-Breslau) (Freyn 1892).

When *Oreoseris nivea* DC. was absorbed into *Gerbera*, the priority was given to *Gerbera* because the latter was the older generic name and, as long as this species stayed in *Gerbera*, the name *Oreoseris* was not available. *Uechtritzia* was described later in 1892; and, as long as *O. nivea* remained in *Gerbera*, then *Oreoseris* continued to be unavailable.

However, as soon as *Gerbera nivea* from Asia was removed from *Gerbera* and a separate genus was formed from the Asian species of *Gerbera* + *Uechtritzia*, then the name *Oreoseris* became available and it is the oldest available name. Hence, these species have been transferred into *Oreoseris*.

### Oreoseris

Taxon classificationPlantaeAsteralesAsteraceae

DC., Prodr. 7(1): 17. 1838.

Onoseris Willd. sect. Isanthus Less., Linnea 5: 338. 1830. Onoseris Willd. subgen. Isanthus (Less.) Less., Syn. Comp.: 119. 1832. Gerbera L. sect. Isanthus (Less.) C.Jeffrey, Kew Bull. 21: 213. 1967.
Gerbera L. sect. Oreoseris (DC.) Sch.Bip., Flora 27: 780. 1844.
Uechtritzia Freyn, Oesterr. Bot. Z. 42(7): 240. 1892. Gerbera sect. Uechtritzia (Freyn) Beauverd, Bull. Soc. Bot. Genève Ser. 2, 2: 43. 1910.

#### Type species.

*Oreoseris nivea* DC., designated by Hansen (1988).

*Oreoseris* has the following 12 species from Eurasia.

### 1. Oreoseris armena

Taxon classificationPlantaeAsteralesAsteraceae

(Freyn et Sint.) V.A.Funk & J.Wen comb. nov.

urn:lsid:ipni.org:names:77176439-1

Uechtritzia armena Freyn et Sint., Oesterr. Bot. Z. 42(7): 241. 1892. Gerbera armena Beauverd, Bull. Soc. Bot. Genève, ser. 2, 2: 43. 1910.

#### Distribution.

Armenia and Turkey.

### 2. Oreoseris delavayi

Taxon classificationPlantaeAsteralesAsteraceae

(Franch.) X.D.Xu & W.Zheng comb. nov.

urn:lsid:ipni.org:names:77176440-1

Gerbera delavayi Franch., J. Bot. (Morot). 2: 68. 1888.

#### Distribution.

China (Guizhou, Sichuan, Yunnan) and N Vietnam.

### 3. Oreoseris gossypina

Taxon classificationPlantaeAsteralesAsteraceae

(Royle) X.D.Xu & V.A.Funk comb. nov.

urn:lsid:ipni.org:names:77176441-1

Chaptalia gossypina Royle, Ill Bot. Himal. 251. T. 59. F. 2. 1835. Gerbera gossypina (Royle) Beauverd, Bull. Soc. Bot. Genève Ser. 2, 2: 40. 1910.
Oreoseris lanuginosa DC., Prodr. 7(1): 17. 1838. Gerbera lanuginosa (DC.) Sch.Bip., Flora 27: 780. 1844.

#### Distribution.

Karakoram, N and C Himalaya.

### 4. Oreoseris henryi

Taxon classificationPlantaeAsteralesAsteraceae

(Dunn) W.Zheng & J.Wen comb. nov.

urn:lsid:ipni.org:names:77176442-1

Gerbera henryi Dunn, J. Linn. Soc., Bot. 35: 511. 1903. Gerbera delavayi var. henryi (Dunn) C.Y.Wu et H.Peng, Acta Bot. Yunnan. 24: 143. 2002.

#### Distribution.

China (Yunnan).

### 5. Oreoseris kokanica

Taxon classificationPlantaeAsteralesAsteraceae

(Regel et Schmalh.) J.Wen & W.Zheng comb. nov.

urn:lsid:ipni.org:names:77176443-1

Gerbera kokanica Regel et Schmalh., Descr. Pl. Nov. Rar. Fedtsch. 53. 1882 (published as Izv. Imp. Obsc. Ljubit. Estesv. Moskovsk. Univ. 34(2): 53. 1882). Uechtritzia kokanica (Regel et Schmalh.) Pobed., Fl. URSS 28: 597. 1963.

#### Distribution.

Pamir-Altai and Tian-Shan regions of C Asia, south to Afghanistan and Kashmir.

### 6. Oreoseris lacei

Taxon classificationPlantaeAsteralesAsteraceae

(G.Watt) V.A.Funk & W.Zheng comb. nov.

urn:lsid:ipni.org:names:77176444-1

Gerbera lacei G.Watt Bull. Misc. Inform. Kew 1911(6): 272. 1911. Uechtritzia lacei (G.Watt) C.Jeffrey, Kew Bull. 21(2): 213. 1967.

#### Distribution.

N India (Himachal Pradesh), S Jammu and Kashmir (Nachar, Baspa, E and NE of Simla, Chamba and Kisthwar).

### 7. Oreoseris latiligulata

Taxon classificationPlantaeAsteralesAsteraceae

(Y.C.Tseng) W.Zheng & J.Wen comb. nov.

urn:lsid:ipni.org:names:77176445-1

Gerbera latiligulata Y.C.Tseng, Acta Bot. Austro-Sin. 3: 11. 1986.

#### Distribution.

China (in Qiaojia county of Yunnan).

### 8. Oreoseris maxima

Taxon classificationPlantaeAsteralesAsteraceae

(D.Don) X.D.Xu & W.Zheng comb. nov.

urn:lsid:ipni.org:names:77176446-1

Chaptalia maxima D.Don, Prodr. Fl. Nepal. 166. 1825. Gerbera maxima (D.Don) Beauverd, Bull. Soc. Bot. Genève Ser. 2, 2: 44. 1910.

#### Distribution.

China (Xizang), Bhutan, India, Nepal, Pakistan and Thailand.

### 9. Oreoseris nivea

Taxon classificationPlantaeAsteralesAsteraceae

DC., Prodr. 7: 18. 1838.

Gerbera nivea (DC.) Sch.Bip., Flora 27: 780. 1844.

#### Distribution.

China (W Sichuan, S Xizang, NW Yunnan), Bhutan, India and Nepal.

### 10. Oreoseris raphanifolia

Taxon classificationPlantaeAsteralesAsteraceae

(Franch.) V.A.Funk & J.Wen comb. nov.

urn:lsid:ipni.org:names:77176447-1

Gerbera raphanifolia Franch., J. Bot. (Morot). 2: 67. 1888.

#### Distribution.

China (NW Yunnan).

### 11. Oreoseris rupicola

Taxon classificationPlantaeAsteralesAsteraceae

(T.G.Gao & D.J.N.Hind) X.D.Xu & V.A.Funk comb. nov.

urn:lsid:ipni.org:names:77176448-1

Gerbera rupicola T.G.Gao et D.J.N.Hind, Fl. China 20–21: 14. 2011.
Gerbera macrocephala Y.C.Tseng, Acta Bot. Austro Sin. 3: 12. 1986, not Gerbera macrocephala Less., Linnaea 5: 295. 1830.

#### Distribution.

China (NW Yunnan).

### 12. Oreoseris tanantii

Taxon classificationPlantaeAsteralesAsteraceae

(Franch.) W.Zheng & X.D.Xu comb. nov.

urn:lsid:ipni.org:names:77176449-1

Gerbera tanantii Franch., J. Bot. (Morot). 7: 155. 1893.

#### Distribution.

China (Yunnan).

## Supplementary Material

XML Treatment for Oreoseris

XML Treatment for Oreoseris armena

XML Treatment for Oreoseris delavayi

XML Treatment for Oreoseris gossypina

XML Treatment for Oreoseris henryi

XML Treatment for Oreoseris kokanica

XML Treatment for Oreoseris lacei

XML Treatment for Oreoseris latiligulata

XML Treatment for Oreoseris maxima

XML Treatment for Oreoseris nivea

XML Treatment for Oreoseris raphanifolia

XML Treatment for Oreoseris rupicola

XML Treatment for Oreoseris tanantii
